# Evaluation of an apparatus to be combined with a smartphone for the early detection of spinal deformities

**DOI:** 10.1186/1748-7161-9-10

**Published:** 2014-07-25

**Authors:** Mark Driscoll, Chanel Fortier-Tougas, Hubert Labelle, Stefan Parent, Jean-Marc Mac-Thiong

**Affiliations:** 1 Spinologics, Montreal, QC, Canada; 2 University of Montreal, Montreal, QC, Canada

**Keywords:** Scoliosis, Early detection, Screening, Smartphone, Angle of trunk inclination, Diagnosis

## Abstract

**Background:**

Mobile smartphones are equipped with inclinometers enabling them to acquire angular clinical measures. The Scolioscreen has been developed in conjunction with a smartphone APP to enable the measure of the angle of trunk inclination (ATI) thus offering a convenient and reliable means to measure and screen for spinal deformities. The objective was to compare the reliability and accuracy of a Scolioscreen-smartphone combination, a smartphone alone, and a Scoliometer, for measuring the angle of trunk inclination in spinal deformities under blinded conditions for intra- and inter-observer analyses.

**Methods:**

A cohort of 39 patients with adolescent idiopathic scoliosis were recruited. Each had maximum ATI measured by 3 observers: attending spine surgeon, nurse, and parent presenting with patient. Two series of measurements were performed by each observer using Scolioscreen-smartphone, smartphone alone and Scoliometer. Intra-class correlation coefficients (ICC) from two-way mixed model based on absolute agreement were used to assess intra- and inter-observer reliability as well as consistency between measurement techniques.

**Results:**

Intra- and inter-observer reliability for measuring maximum ATI was 0.94-0.89 with Scolioscreen-smartphone, decreased to 0.89-0.75 for smartphone alone, and was 0.95- 0.89 for Scoliometer. Considering Scoliometer measurement taken by surgeon the gold standard, there was excellent consistency with measurements from Scolioscreen-smartphone taken by surgeon (ICC = 0.99), nurse (ICC = 0.95), and parent (ICC = 0.91). Conversely, consistency decreased when surgeon (ICC = 0.86), nurse (ICC = 0.86) and parent (ICC = 0.85) used smartphone alone.

**Conclusion:**

Study shows the Scolioscreen-smartphone to overcome limitations associated with ATI measurements using smartphones alone. The Scolioscreen-smartphone provides a reliability and consistency similar to the gold standard (use of Scoliometer by spine surgeon) and enables a parent to take reliable measurements on their own thus offering an accessible and convenient tool for all to use.

## Background

Adolescent idiopathic scoliosis (AIS) defines a spinal deformity of unknown cause with a prevalence of 2-4% and most often presents in young females aged 10 to 12 years. Treatment of scoliosis is determined by the severity of the curvature. Typically, brace treatment is initiated after a lateral curve of at least 20–25 degrees is detected in immature patients. Subsequent progression of the curve to magnitudes greater than 40–45 degrees will entail the suggestion of a surgical intervention in selected patients.

Early detection of scoliotic deformities remains controversial on the premise that the ultimate goal of reducing surgical intervention is curtailed by disputes surrounding the effectiveness of early treatment methods such as bracing. Subsequently, the United States have limited school screening programs while Canada has discontinued such efforts [1]. In 2010, the Scoliosis Research Society (SRS) formed an international task force mandated to analytically explore the merit of scoliosis screening programs. These efforts led to the consensus that scoliosis screening is recommended as a valuable tool to detect deformities of Cobb angles 10 degrees or greater which should be referred for diagnostic evaluations [2]. Speaking to early treatment effectiveness, the SRS task force also point out that level I evidence now exists to support brace treatment in AIS as a result of the recently published BrAIST multicenter NIH trial [3]. Consequently, this study is now an important feature in advocating for the adoption of early detection of scoliosis through the assessment of the angle of trunk inclination.

The gold standard of AIS screening is to use a Scoliometer (Orthopedic Systems Inc., Hayward, CA) in combination with an Adams Forward bending test to detect trunkal asymmetry as portrayed through the measured angle of trunk inclination (ATI). The Scoliometer is a medical device providing a measure of inclination derived from the relative calibrated position of an air bubble traveling within a vial of encased liquid. The SRS task force suggested that a measure between 5 and 7 degrees be used as the screening threshold while evaluation be performed for females twice at 10 and 12 years of age and once for males 13 or 14 years of age [4].

**Figure 1:**
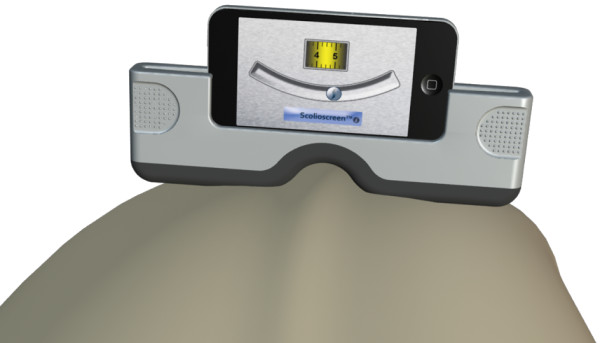
Demonstration of the Scolioscreen-smartphone combination positioned on a patients back during an Adams test.

Scoliosis screening programs are conventionally reserved to health care professionals and are not widely practiced. This is perhaps related to general lack of knowledge of the public as well as the Scoliometer being focused as a clinical tool. Today’s technological advancements have integrated inclinometers into smartphones which enable them to effectively take measures of inclination, similar to the Scoliometer. Currently, several smartphone applications, otherwise known as Apps, suggest to provide measures of ATI. However, the smartphones enabling these Apps lack several characteristics of the Scoliometer and have not been validated as providing reliable clinical measures. Two physical characteristics may impede measures of ATI taken with a smartphone. First, a smartphone is not wide enough to span the ribs of the patient from which the ATI measure is derived. Second, the smartphone is not adapted to conform to the protrusion provided by the spinal process of the patient. Consequently, the widespread convenience of using one’s smartphone as a scoliosis screening tool has an appeal that inspired the development of the Scolioscreen (Spinologics Inc., Montreal, Canada).The Scolioscreen was developed as a medical device to be used in combination with a smartphone to screen for scoliosis (Figure 1). The Scolioscreen is made from a medical grade thermoplastic rubber and sized to effectively hold all smartphones, with or without a protective case, and designed to mimic the undersurface of a Scoliometer. In theory, the Scolioscreen would offer a much more accessible and convenient means to screen for and monitor scoliosis, in line with newer technologies developed for smartphones. However, in order to confidently advocate the performance of the Scolioscreen, its intra- and inter-observer reliability and accuracy as compared to the gold standard, the Scoliometer, was required. Thus the purpose of the study was to evaluate the efficacy of the Scolioscreen-smartphone combination and smartphone alone as compared to the Scoliometer. Furthermore, to encourage the widespread adoption of this medical tool, the study was performed by spine surgeons, a nurse, and the parent of the presenting patient.

## Methods

**Figure 2:**
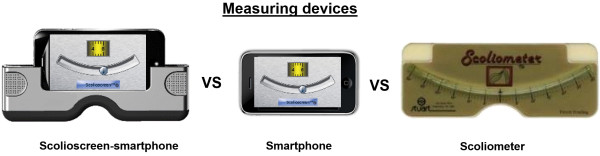
Measuring devices analysed and compared.

The protocol for subject recruitment and consent was approved by the institutional ethical committee at the Sainte-Justine university hospital center. Subject recruitment took place during scheduled appointments at a scoliosis clinic in a pediatric hospital setting. Patients aged between 10 and 18 years and presenting with an adolescent idiopathic scoliosis were solicited to be included in the study. Patients were excluded if presenting with a leg length discrepancy of more than 2 cm, having underwent lower limb or spinal surgery of any kind other than scoliosis surgery or having any other disease affecting posture or trunk shape. Patients were also excluded if their parent was absent during the appointment.Three users, a spine surgeon, a nurse, and the patient’s parent each evaluated three measuring devices, the Scolioscreen-smartphone combination, the smartphone alone, and the Scoliometer (Figure 2). The smartphone used in the study was consistently an iPhone 4 dimensioned as 115.2 mm long, 58.6 mm wide and 9.63 mm thick (Apple Inc., Cupertino, CA) and the inclinometer hardware used was the Scoligauge APP. The smartphone inclinometer is known to have an inclinometer accuracy of the range of ± 0.1 degrees. The Adam Forward Bending test position was used in all measures where patients were asked to bend forward with palms together in order to measure the ATI. The patient’s ATI values were measured by the nurse and parent before the arrival of the spine surgeon using all three measuring devices. Prior to the measurement sessions, the nurse explained to the parent how to properly use the three measuring devices emphasizing that they hold the measuring device perpendicular to the length of the spine and moving along and centered to the spinous processes. The spine surgeon then measured a first series of ATI values using all three measuring devices at the beginning of the consult and a second series at the end, with an average of 10 minutes between.

Each measurement series of ATI was performed in a random order of the measuring devices. Moreover, all measures were performed blind by all users (spine surgeon, nurse, and parent). Two series of measurements were performed by each user. The measuring devices were positioned facing away from user while a research assistant was the only one able to see the measured values and took note of the corresponding maximum ATI measured with each measuring device.

Statistical analyses were assessed by using descriptive statistics for all data processing. Intra-class correlation coefficients (ICC), having an associated 95% confidence interval, was utilized to analyze the inter- and intra-observer reliability. The ICC used a 2-way mixed model on absolute agreement. All statistical analyses were performed with IMB SPSS Statistics 20.0 software (IBM Armonk, New Corporation, Armonk, New York, U.S). Strength of the reliability results were assessed using recommendations associating values of 0–0.20, 0.21-0.40, 0.41-0.60, 0.61-0.80, and 0.81-1.0 with slight agreement, fair agreement, moderate agreement, substantial agreement, and almost perfect agreement respectively [5].

## Results

Eight boys and 31 girls composed the study sample. The mean age of the cohort was 16 years old (SD, 1.4). Eight participants had undergone prior spinal surgery to correct their scoliosis. The mean magnitude of curves measured by Cobb method was 20.7 degrees (SD, 15.2°) for thoracic curves and 19.9 degrees (SD, 8.5°) for lumbar curves. The curve magnitudes varied between 3–58 degrees. The mean angle measurements for each measuring device taken by all users are outlined in Table 1. The gold standard being the spine surgeon having taken the ATI measurement with the Scoliometer was 6.95° (SD, 4.59). The average score taken by the spine surgeon with the smartphone was 7.59 (SD, 5.95) and with the Scolioscreen-smartphone combination was 7.05 (SD, 4.81).

**Table 1:** Mean measures taken by users using various measuring devices

	**Measuring device**					
	**Solioscreen-smartphone**		**Smartphone**		**Scoliometer**	
**User**	**Mean (°)**	**SD**	**Mean (°)**	**SD**	**Mean (°)**	**SD**
Spine surgeon	7,05	4,81	7,59	5,95	6,95	4,59
Nurse	7,19	5,48	7,98	6,39	7,73	4,77
Parent	6,39	4,82	5,86	5,06	6,75	4,73

Table 2 details the calculated ICC values in comparison to the gold standard. The spine surgeon achieved an ICC of 0.86 and 0.99 when using the smartphone and Scolioscreen-smartphone combination respectively. Similarly, the nurse ICC scores where 0.86 with the smartphone and 0.95 with the Scolioscreen-smartphone. The parent ICC scores where 0.85 with the smartphone and 0.91 with the Scolioscreen-smartphone combination.

**Table 2:** Calculated ICC values as a function of the gold standard (spine surgeon using the Scoliometer)

**User**	**Measuring device**	**ICC value**
Spine surgeon	Smartphone	0.86
	Scolioscreen-smartphone	0.99
Nurse	Smartphone	0.86
	Scolioscreen-smartphone	0.95
Parent	Smartphone	0.85
	Scolioscreen-smartphone	0.91

Table 3 reports the intra- and inter-observer ICC values calculated between measures taken by the spine surgeon and other users. The intra-observer reliability for measurements acquired by all users were 0.95, 0.89, and 0.94 taken with the Scoliometer, smartphone, and Scolioscreen-smartphone combination respectfully. The inter-observer results were 0.89 for the Scoliometer, 0.75 for the smartphone, and 0.89 for the Scolioscreen-smartphone combination.

**Table 3:** Intra- and inter-observer ICC values calculated from measures taken by the spine surgeon and other users

**Measuring device**	**Intra-observer ICC**	**Inter-observer ICC**
Scoliometer	0.95	0.89
Smartphone	0.89	0.75
Scolioscreen-smartphone	0.94	0.89

## Discussion

This study demonstrates the reliability and accuracy of a new medical device for measuring the angle of trunk inclination when screening patients with spinal deformities. Perhaps most interestingly, this study reported that a parent of a child having a spinal deformity and using the Scolioscreen-smartphone combination is as accurate as a spine surgeon using the gold standard, the Scoliometer. To the authors’ knowledge, this is the first study to report the performance of a medical device for combination with a smartphone, the Scolioscreen, to provide an accurate and convenient platform for screening patients with a spinal deformity.

Various smartphone Apps currently exist that seek to mimic the angular measuring capacity of the Scoliometer. A recent study showed the measuring reliability of such a smartphone App when positioning the smartphone running the App on the top flat surface of the Scoliometer to verify if it provided angle readings in agreement with the Scoliometer [6]. However, this study only evaluated the angular accuracy of the App while no actual patient measures were acquired leaving the clinical value of using only a smartphone in question. Another study, reported that an acrylic sleeve, adapted to hold a smartphone and shaped like a Scoliometer, showed positive inter- and intra-observer reliability [7]. The authors also opined that the use of a smartphone alone would not suffice as a screening device due to the differences with the Scoliometer. This study was also not performed with real patients but used plaster torso molds to represent a patient in an Adams bending position. Moreover, the proposed acrylic sleeve does not provide a secure fit of the smartphone within the device which may lead to movement therein and subsequent measurement errors when used with a real patient. The Scolioscreen, reported herein, enables a “one size fits all” for any smartphone thus securing the phone within the device while taking measures on a patient and possibly contributing to its strong reliability. But more importantly, the Scolioscreen adopts the same geometry as the Scoliometer on its undersurface in order to conform with the protruding spinous processes and to rest securely on the rib cage bilaterally.

This study was performed on patients having either unoperated or operated scoliosis deformities. Special care was taken to blind the users while taking the measures. Patients were provided appropriate time lapses between measurements to avoid discomfort or any fatigue which may lead to discrepancies between measurements. It is expected that the high intra-observer reliability using the Scoliometer or the Scolioscreen-smartphone combination be consistent irrespective of the time between measurements provided that all measures were taken blinded. The least experienced user, the parent of the patient, was provided instructions in person based on the detailed instructions of use and instructional videos provided with the Scolioscreen. The adoption of medical professionals to the Scolioscreen should be seamless and convenient while the congruence between the methods of use of the Scoliometer and Scolioscreen should continue to ensure measurement accuracy as reported in this study.

The current tendency of medical professional to advocate the value of early detection of scoliosis corresponds well with new class I evidence in support of the effectiveness of bracing [3]. Furthermore, new fusionless treatments of progressive scoliosis are in development showing encouraging clinical outcomes [8-10]. It may be likely that one day fusionless treatments will offer an early alternative for patients showing detectable signs of progression. Thus, AIS detection methods and screening processes go hand in hand with successful administration of these early treatments and towards the improved care or AIS patients. Remaining consistent with the recommendation of the SRS taskforce, upon using the Scolioscreen-smartphone combination, the authors suggest that a threshold of 5–7 degrees be used for the early detection of scoliosis.

## Conclusion

This study demonstrates the shortcomings and lack of reliability when using only a smartphone to attempt to measure the ATI. Therefore, the authors do not recommend using a smartphone alone when screening for scoliosis. Further, this study confirms the Scolioscreen-smartphone combination provides reliability and consistency similar to the gold standard (Scoliometer used by a spine surgeon) and enables a parent to take accurate measurements on their own thus offering an accessible and convenient tool for all to use.

In conclusion, this study shows that parents and clinicians should strongly consider adding the Scolioscreen when using a smartphone to assess the ATI and screen for scoliosis if a reliability similar to that obtained with the Scoliometer is desired.

### IRB approval

This study was approved by the institutional ethical committee.

## Competing interests

Mark Driscoll, Stefan Parent, Hubert Labelle, and Jean-Marc Mac-Thiong are shareholders at Spinologics Inc. Mark Driscoll, Stefan Parent, Hubert Labelle, and Jean-Marc Mac-Thiong are inventors on pending patents of the apparatus disclosed herein.

## Authors’ contributions

MD, CFT, SP, HL, JMMT: contributed to the conception and design of the study. CFT, SP, HL, JMMT: contributed to the acquisition of data. MD, CFT, SP, HL, JMMT: contributed to the analysis of data. MD, CFT, SP, HL, JMMT: contributed to the drafting, revision, and have provided final approval of the manuscript. All authors read and approved the final manuscript.

## References

[B1] Canadian Task Force on the Periodic Health Examination The periodic health examination Can Med Assoc J 1979 121 1193 1254 115569 PMC1704686

[B2] Labelle H Richards SB De Kleuver M Grivas TB Luk KD Wong HK Thometz J Beauséjour M Turgeon I Fong DY Screening for adolescent idiopathic scoliosis: an information statement by the scoliosis research society international task force Scoliosis 2013 8 1 17 [Epub ahead of print] 10.1186/1748-7161-8-17 24171910 PMC3835138

[B3] Weinstein SL Dolan LA Wright JG Dobbs MB Effects of bracing inadolescents with idiopathic scoliosis N Eng J Med 2013 369 1512 1521 10.1056/NEJMoa1307337 PMC3913566 24047455

[B4] Richards BS Vitale MG Statement: screening for idiopathic scoliosis in adolescents: an information statement J Bone Joint Surg Am 90 1 198 10.2106/JBJS.G.01276 18171974

[B5] Landis JR Koch GG The measurement of observer agreement for categorical data Biometrics 1977 33 159 174 10.2307/2529310 843571

[B6] Franko O Bray C Newton P Validation of a scoliometer smartphone APP to assess scoliosis J Pediatr Orthop 2012 32 8 e72 e75 10.1097/BPO.0b013e31826bb109 23147635

[B7] Izatt M Bateman G Adam C Evaluation of the iPhone with an acrylic sleeve versus the Scoliometer for rib hump measurement in scoliosis Scoliosis 2012 7 14 10.1186/1748-7161-7-14 22846346 PMC3479427

[B8] Driscoll M Aubin Moreau A Wakula Y Maini S Parent S Novel hemi-staple for the fusionless correction of pediatric scoliosis: influence on intervertebral discs and growth plates in a porcine model J Spinal Disord Tech Epub ahead of print 10.1097/BSD.0b013e31828b2f15 27755203

[B9] Betz R Andrea L Mulcahey M Chafetz R Vertebral body stapling procedure for the treatment of scoliosis in the growing child Clin Orthop Relat Res 2005 434 55 60 15864032 10.1097/01.blo.0000163472.46511.a8

[B10] Braun J Akyuz E Ogilvie J Bachus K The efficacy and integrity of shape memory alloy staples and bone anchors with ligament tethers in the fusionless treatment of experimental scoliosis J Bone Joint Surg Am 2005 87 9 2038 2051 10.2106/JBJS.D.02103 16140820

